# Epigenetic Enzyme Mutations: Role in Tumorigenesis and Molecular Inhibitors

**DOI:** 10.3389/fonc.2019.00194

**Published:** 2019-03-29

**Authors:** Mei Han, Lina Jia, Wencai Lv, Lihui Wang, Wei Cui

**Affiliations:** Department of Pharmacology, Shenyang Pharmaceutical University, Shenyang, China

**Keywords:** DNMT, mutation, small molecule inhibitors, tumor, histone modification enzyme

## Abstract

Epigenetic modifications, such as DNA methylation and histone modification, result in heritable changes in gene expression without changing the DNA sequence. Epigenetic regulatory enzymes such as DNA methyltransferases, histone methyltransferases, and histone deacetylases are involved in epigenetic modification. Studies have shown that the dysregulation caused by changes in the amino acid sequence of these enzymes is closely correlated with tumor onset and progression. In addition, certain amino acid changes in the metabolic enzyme isocitrate dehydrogenase (IDH) are linked to altered epigenetic modifications in tumors. Some small molecule inhibitors targeting these aberrant enzymes have shown promising anti-cancer efficacy in preclinical and clinical trials. For example, the small molecule inhibitor ivosidenib, which targets IDH1 with a mutation at R132, has been approved by the FDA for the clinical treatment of acute myeloid leukemia. In this review, we summarize the recurrent “hotspot” mutations in these enzymes in various tumors and their role in tumorigenesis. We also describe candidate inhibitors of the mutant enzymes which show potential therapeutic value. In addition, we introduce some previously unreported mutation sites in these enzymes, which may be related to tumor development and provide opportunities for future study.

## Introduction

The term “epigenetics” describes inheritable changes of gene expression with no alteration of the DNA sequence (1). As the field of epigenetics has expanded, the connection between epigenetic changes and the occurrence and development of tumors has received more attention (2). The structure of chromatin is the basis for modulating gene expression: euchromatin has an open structure that is typically associated with active transcription, while heterochromatin is tightly compacted and usually associated with transcriptional repression. Epigenetic modification such as DNA methylation and histone modification are important for regulating chromatin structure and therefore gene expression. These modifications are catalyzed by epigenetic regulatory enzymes, including DNA methyltransferases, histone methyltransferases and histone deacetylases.

Recent studies have shown that the dysregulation (e.g., overexpression) of these enzymes plays a crucial role in tumorigenesis. Some small molecule inhibitors targeting these aberrantly expressed epigenetic regulatory enzymes have been approved by the FDA for the treatment of certain cancers. For example, the small molecule inhibitor 5-azacytidine, which targets the DNA methyltransferase DNMT3A, has been approved for clinical treatment of patients with acute lymphoblastic leukemia (AML) (3), and belinostat, which targets histone deacetylases (HDACs) in peripheral T-cell lymphoma (PTCL), was approved for use in 2014 (4). In addition, the inhibitor EPZ6438, which targets EZH2, a histone methyltransferase, has been approved for testing in the clinic (5).

In recent years, increasing evidence has shown that epigenetic regulatory enzymes are mutated in various types of cancer, and mutations of these enzymes are closely related to the malignant phenotype (6, 7). Hence, inhibitors that target these mutant enzymes have gradually entered preclinical and clinical research. In this review, we first summarize the epigenetic regulatory enzymes and their mutations in different types of tumors, and then we explain how the mutations are correlated with tumorigenesis. Finally, we present some small molecule inhibitors which target epigenetic regulatory enzymes, especially their mutated forms, and may have potential therapeutic value in the future.

## DNMTs and Their Mutations in Cancer

### DNMTs in Cancer

DNA methylation, which is one of the major epigenetic regulatory mechanisms, plays a crucial role in many life processes (8). In eukaryotic cells, DNA methylation is a stable gene silencing modification that is copied during DNA replication (9). DNA methylation predominately occurs at cytosine residues in 5′-CpG-3′ dinucleotides, with S-adenosyl methionine (SAM) as the methyl donor (10). In mammals, DNA methylation is catalyzed by enzymes in the DNA methyltransferase (DNMT) family, mainly DNMT1, DNMT3A, and DNMT3B. DNMT1 maintains the methylation status of newly replicated (hemi-methylated) DNA, whereas DNMT3A and DNMT3B are responsible for *de novo* DNA methylation (11). The mechanism by which DNA methylation regulates gene expression involves blocking the binding of transcription factors to DNA and the recruitment of proteins containing a methylated CpG-binding domain to inhibit gene expression in tumor cells (12). The methylation profiles in different cells are not the same, and this has functional consequences. In normal cells, gene promoters containing CpG islands are usually unmethylated, which maintains the chromatin in an open structure, and hence enhances the transcription of the gene. However, in tumor cells, the CpG island-containing promoters of tumor suppressor genes are usually methylated, and thus the euchromatin is converted to compacted heterochromatin (13). These findings indicate that DNA methylation regulates tumorigenesis and progression by inhibiting the expression of tumor suppressor genes.

### DNMT Mutations in Cancer

Recently, studies have shown that mutations of DNMT family, especially DNMT3A, are prominent features of many tumors and can lead to malignant transformation (14). DNMT3A is one of the most frequently mutated DNA methyltransferase in AML (6) and myelodysplastic syndromes (MDS) (15). Some reports have shown that mutations in DNMT3A are present in up to 20% of AML cases and are associated with poor prognosis (8, 16). Although a large number of mutations in the DNMT3A have been reported, ~50% of the changes are in the catalytic domain at position R882 (most commonly R882H) (8, 17, 18). Table 1 shows DNMT3A mutations, including hotspots and non-reported mutation sites, in various tumors. In addition, mutations in DNMT1 have been described in colorectal (29), prostate and hematological malignancies (30). The gene encoding DNMT3B was reported to be mutated in immunodeficiency syndrome, but mutations have rarely been reported in tumors (31). In addition, except DNMTs' mutations in various cancers, DNA hydroxymethylase TET2, which catalyzes the conversion of 5-methyl-cytosine to 5-hydroxymethyl-cytosine, has been reported in recent years for its mutations in various diseases, especially AML and MDS (32). The above results indicated that the mutations in DNMT and its related enzyme are frequent, which suggesting the potential role of them in tumorigenesis.

**Table 1:** Epigenetic regulatory enzymes mutation sites and their function in different types of cancer.

**Enzyme**	**Gain-of-function mutation**	**Function of mutation**	**Non-reported mutation**	**Cancer type (references)**	**Inhibitor**
DNMT3A	R882(H/C/P); R882C	Migration; Proliferation; Colony formation; Blocking differentiation	Y735F, V716F, R729(Q/H/W), R803S, R736H, K829R, P718L, C497Y, D781G, G646V, A741V, F909C, M801V; R792H, G26V, S708C, G412W, A254P, E629D, G293W, V763F, R771Q, Y533C, Q485H, K680R, S878P, E725V, R209P, P59L, R379L, M864I, R326P, P804S, V258M, W327G, C494S, S312F, D781H, G413S, S669C, A116S, F909S, R458Q, R55H, Y724C, V563M, D857V, W795C, P89R, D618N, Y735C, V560L, G570W, M78I, D279V, E392D, M224V, Q248R, V895M, V401L, G685V, C559Y, E854Q, G49R, G890C, E323K, P709L, Y359C, E213D, G746V, P58L, R885S, V687F, P425T	**Leukemia (****6**, **8**, **16****)** Lung cancer	5-azacytidine; Dichlone; SYC-52221; EPZ004777; EPZ5676; (pan-inhibitors)
EZH2	Y641(S/H/F/N), A687V, A677G, Y646H; Y641(S/H/N/F), Y646(S/H); Y646H, A687V	Migration; Proliferation; Tumor growth; Poor prognosis;	E740K, R679H, G159R, N670K, S271F, W113C, K660R, K660E, D185H, T53M, D183E, M701V, Y447H, V702G, C642S, T144I, E636D, R685H, D659A, F672L; S533L, R342Q, R216(Q/W), P132S, P219S, G2(D/S), D316G, R34P, P746S, S229L, S405L, T4(I/P), D142V, A226V, S228F, P426S, R355G, C530W, G704S, G459E, R81S, P417Q, R456S, P535H, R382M, P262H, P631H, L338F, S474F, E391K, P527L, N366S, K510R, V675M, D511N, A590V, H521Y, I651T; K426Q, S652(C/F), P493A, I633M, T467P, S624C, K550T, I150V, E173Q, H129D, Q653E, K545T, L315V, Q648E, S647C; K510R, E374Q, Q548E, N310S, A340T, P262(L/T/I), R685G, E650Q, R308L, A715V, A622, R497Q, D233Y, R34L, E341K, R64M, D186N, K39E, H613Q, S647C, Q66R, R357L, E312K, Q94R, P481S, F667L, H502Q, R52I, G5R, S647F, R527W,S40C	**Lymphoma (****19****–****21****)** **Melanoma (****22****)** Breast cancer Lung cancer	EPZ005687; GSK126; EPZ-6438; CPI-1205; (pan-inhibitors)
IDH1	R132(C/S/G/H/L); R132(C/H); R132(C/G/H); R132(C/S/G/L); R132(C/G/L); R132(C/H); R132(C/L)	Proliferation; Migration; Colony formation; Blocking differentiation; Angiogenesis; Inhibition of apoptosis	G339E, G161R; V178I, G370D, S210N, R20Q; R119Q, E306D, T106M; N349S; C297S, Q198P, N171T, M182V; D160E, D79N, T327A, G263V, L88F, K217N, Y235C, R338T, K81N, K151N, A179D, I189V, E84Q; R49C, M290I, Q228R, E361K, E360K, S94P, A341V, G339W, G284V, A282V; I102T, E28Q, R109K, D375Y, A179D, Y34C, I333V, V294L, I189F, A193S, D299E, G175V, G221(L/V/W), L359F, E262Q, K406E T302R, R119W, G310V, I380F, N961	**Glioma (****7**, **23****)** **Lymphoma (****24****)** **Prostate cancer (****25****)** **Cholangiocarcinoma (****26****)** **Hepatic carcinoma (****27****)** Breast cancer Melanoma Lung cancer	Ivosidenib (specific targeting mutant IDH1 in R132); FT-2120 (specific targeting mutant IDH1 in R132); IDH-305 (specific targeting mutant IDH1); AGI-881 (pan-inhibitor)
DH2	R172(S/K/G/W/M); R172(K/G/M/S); R172K; R172(K/S); R172K	Proliferation; Migration; Colony formation; Blocking differentiation; Angiogenesis;	G383V, K251N; H430Y, R140(Q/L/G), D225N, N156I; I62V, I61V; S301L, N156S, R60G, F270S, I419T; R122(C/S), I139F, G137E, G387W, E68K, M248I, P198T, G176N, A321V, M221I, R362W; R188W, H273D, Q267E, S249G, Q322K, I62M; I240V, F192L, T146S, E345K;	**Glioma (****7**, **28****)** **Lymphoma (****24****)** **Prostate cancer (****25****)** **Hepatic carcinoma (****27****)** Melanoma Lung cancer Breast cancer	Enasidenib (specific targeting mutant IDH2); AGI-881(pan-inhibitor)
HDAC2	–	–	Q354L, D175N, D231H, S118P, K466Q, N470I; M65V, G28R, A257T, P102A, R78(L/W), L42F, D456N, A120V, C101F, E109Q, A122S, D426Y	Leukemia Lung cancer	–

*Publications reporting cancers with the indicated gain-of-function mutations are in bold. Non-reported mutations in the same proteins were identified from the cBioPortal website*.

### Function of DNMT Mutations

Mutations in DNMTs are closely correlated with the biological characteristics of malignant tumors and they increase the ability of cancer cells to undergo proliferation, migration, colony formation, and self-renewal. Recently, the relationship between the DNMT3A R882C mutation and the migration of tumor cells has been investigated *in vitro* (33). The results showed that the OCI-AML3 cell line, which carries the R882C mutation, had a greater migration ability than cell lines carrying wild-type (WT) DNMT3A, and infiltrated into the meninges of mice after intravenous infusion. This indicates that the DNMT3A R882 mutation contributes to the enhanced migration of malignant cells. It was also shown that the DNMT3A R882H mutation increases the proliferative capacity of hematopoietic cells and actively promotes the growth of monocytes and macrophages (33). Mechanistically, DNMT3A R882 mutant proteins interact with polycomb repressive complex 1 (PRC1) to block the differentiation of hematopoietic stem cells and lymphocytes by down-regulating differentiation-associated genes (34). Furthermore, cells with DNMT3A R882 mutations have a higher colony forming capacity than WT cells (34). In addition, it was reported that DNMT3A R882 mutations may induce chemotherapy resistance in AML patients. Guryanova et al. reported that the DNMT3A R882H mutation increases the risk of AML patients being resistant to anthracycline therapy by dysregulating nucleosome remodeling (35). Some reports have shown that the DNMT3A R882 mutation was negatively correlated with the prognosis of AML patients. The 5-year overall survival of AML patients with DNMT3A mutations was significantly shorter than AML patients without such mutations (36, 37). Accordingly, Delhommeau et al. reported that TET2 mutations are early events in patients with some MDS and secondary AML and confirmed the important role of TET2 in maintaining the balance between hematopoietic cell survival, growth and differentiation (38). Studies have shown that leukemia-associated missense mutations impair the enzymatic activity of TET2 and lead to a decrease in the genomic level of 5-hydroxymethyl-cytosine, which disrupts normal hematopoiesis and may accelerate leukemia formation (32). All of the above observations show that mutations in DNMT3A and TET2, to some extent, promote oncogenesis, and tumor progression.

## HMTs and Their Mutations in Cancer

### HMTs in Cancer

Histone methylation is involved in the regulation of various biological processes such as gene expression, DNA repair, differentiation, replication and growth (39). Histone methyltransferases catalyze the transfer of the methyl group of SAM to histone arginine or lysine residues. A number of HMTs have been identified, including histone lysine methyltransferases (HKMTs) and histone arginine methyltransferases (HRMTs), which have specific substrates and residues. EZH2 belongs to the HKMT family and is frequently overexpressed in various cancerous tissue types such as breast, prostate and lung (19, 20, 40).

### HMT Mutations in Cancer

EZH2 is a histone methyltransferase that catalyzes the trimethylation of arginine 27 in histone H3 (H3K27). Reports of EZH2 mutations in cancer have increased in recent years. Mutations in epigenetic regulatory enzymes are either gain-of-function or loss-of-function (3). EZH2 gain-of-function mutations were previously reported in lymphoma, and the probability of EZH2 mutation in melanoma was recently reported to be about 2%. Popov et al. found that 27% of follicular lymphoma cases had EZH2 mutations at 3 recurrent hotspots (Y646, A682, and A692) (24). Other gain-of-function hotspot mutations including Y641, A677, and A687 in the catalytic SET domain of EZH2 are prevalent, accounting for ~10–24% of non-Hodgkin's lymphoma (26). In addition to these hotspot mutations, we have summarized some non-reported mutation sites that have yet to be studied, as shown in Table 1.

### Function of HMT Mutations

The dysregulation of H3K27 trimethylation (H3K27me3) is important in human tumorigenesis (25), and some reports have shown that mutant EZH2 increases the level of H3K27me3 in follicular lymphoma, germinal center B-cell type diffuse large B-cell lymphomas (21, 24, 41) and metastatic skin melanoma (42). The level of H3K27 monomethylation and dimethylation in cancer cells and tumor tissues with heterozygous EZH2 mutations at Y641 and A677 is decreased, while the level of H3K27 trimethylation is increased, resulting from the changed substrate preference of the mutant enzymes (22, 41). Barsotti et al. revealed that cells with a gain-of-function EZH2 mutation at Y641 displayed enhanced motility compared to control cells, forming highly dynamic collective migrating chains under 3D culture conditions (42). The Y641 EZH2 gain-of-function mutant cells also had a significant growth advantage in melanoma xenografts. Others have reported that mutations in EZH2 can promote lymphocyte proliferation and maintain the enhanced histone methyltransferase activity *in vivo*, subsequently increasing tumorigenicity (26). Somatic mutations in EZH2 have been shown in many reports to correlate with poor prognosis in patients with AML and myeloproliferative neoplasms (6, 43). Thus, mutations in EZH2 may contribute to the enhancement of the malignant phenotype.

## HMT-Related Enzymes and Their Mutations in Cancer

### HMT-Related Enzymes in Cancer

Isocitrate dehydrogenase (IDH) plays a key role in the Kreb's cycle, catalyzing the conversion of isocitrate into α-ketoglutarate (α-KG). The two major human IDH proteins, IDH1 and IDH2, are not HMTs, but their mutant forms indirectly contribute to effects on histone methylation by catalyzing the conversion of α-KG to 2-hydroxyglutarate (2-HG). Accumulation of 2-HG can inhibit the activity of a broad range of histone demethylases, inducing hypermethylation which is observed in certain cancers such as gliomas (44). In addition, high levels of 2-HG can inhibit α-KG-dependent prolyl hydroxylase, which is important for the degradation of hypoxia-inducible factor (HIF)-1α, a regulator of histone demethylases (7, 23, 28). Mutated forms of IDH therefore mimic the effects of HMTs.

### HMT-Related Enzyme Mutations in Cancer

As mentioned above, specific mutants of IDH can catalyze the conversion of α-KG to 2-HG, and 2-HG inhibits not only histone demethylases but also TET DNA demethylases. This can cause increased methylation of both DNA and histones (3). Therefore, mutant IDH may be an oncoprotein and 2-HG may be an “oncometabolite” (7). In recent years, hotspot mutations in IDH1/2 have been reported in various tumors (Table 1). It has been reported that mutations of IDH1 and IDH2 occur in the vast majority of low-grade gliomas and secondary high-grade gliomas, and also in some cases of AML (27). In addition, IDH mutations have been found in solid tumors such as cholangiocarcinoma and prostate cancer (45, 46). The hotspot mutation of IDH1 is located at R132, while the hotspot mutation of IDH2 is located at R172, which is homologous to R132 in IDH1. We also found that other mutations of IDH1, including G339(E/W), R49C, R119(Q/W), and V294L, may be hotspot mutations in various tumors (Table 1). In addition to mutations in the enzyme of IDH family in the Kreb's cycle, two other metabolic enzymes involved in epigenetic regulation, SDH and FH, have also been reported in recent years to mutate in germline frequently. Ciccarone et al. concluded that SDH mutations in germline are responsible for the formation of hereditary paragangliomas and adrenal gland pheochromocytoma, whereas FH mutations are typical of hereditary leiomyomatosis and renal cell cancer (HLRCC) (47).

### Function of HMT-Related Enzyme Mutations

**Figure 1:**
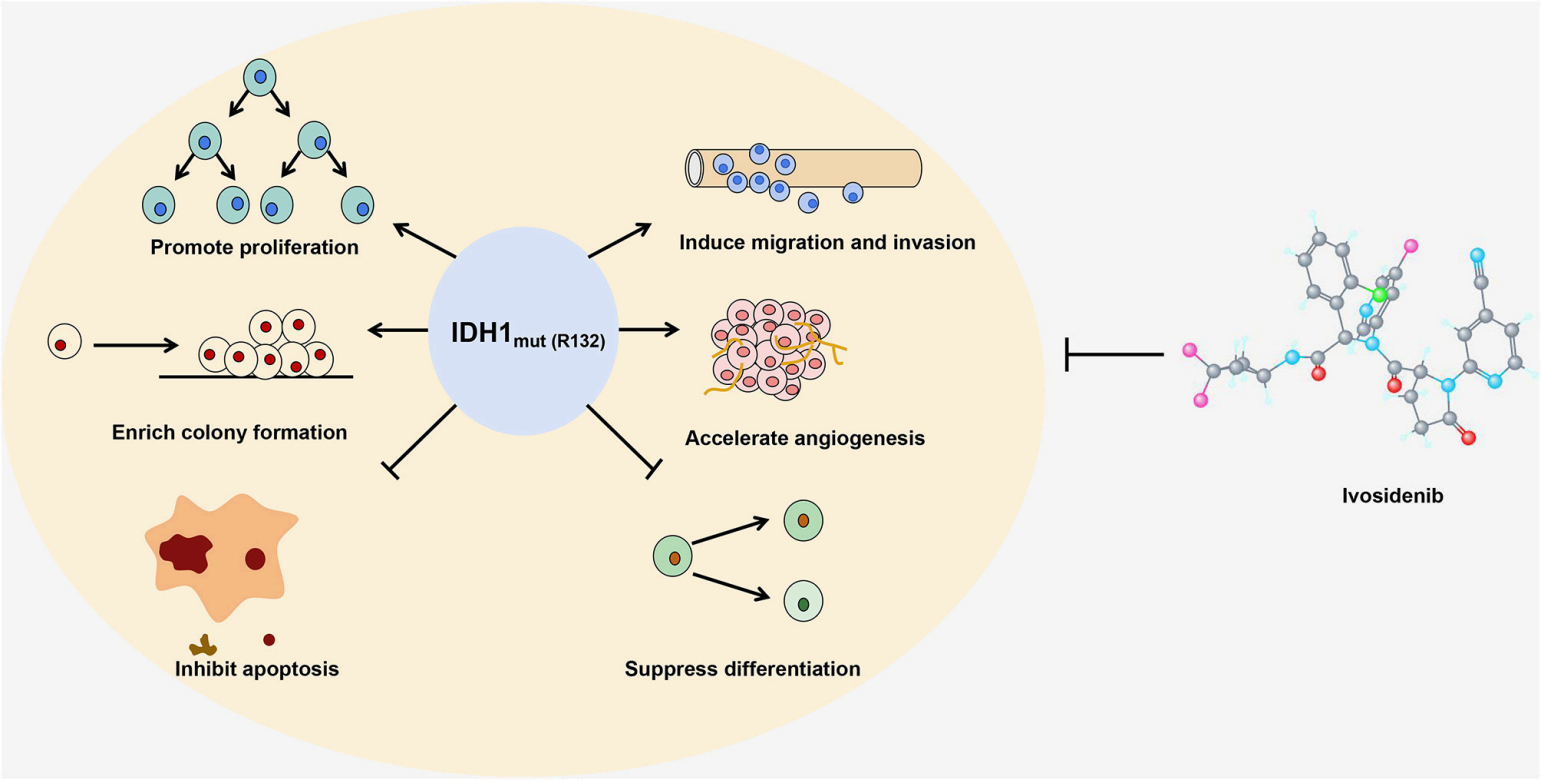
The IDH1 R132 mutant is shown as an example to illustrate how a gain-of-function mutation in an epigenetic enzyme affects the growth and differentiation of cells. Ivosidenib, a specific inhibitor of the IDH1 R132 mutant, is shown at the right.

Several groups have investigated the effect of IDH hotspot mutations, which mimic the activity of HMTs, in cancer. Cohen et al. elucidated that mutant IDH can trigger tumorigenesis. In detail, they found that somatic mutations in IDH1 at R132 or IDH2 at R172 led to increased risk of glioma, hemangiomas and chondrosarcoma, and they demonstrated that the mutated IDH contributed to the increased cell proliferation, colony formation, and inability to differentiate (7). In addition, Fu et al. showed that the IDH2 R172 mutation accelerated the migration and growth of C6 glioma cells by increasing the stability of HIF-1α (48). They also reported that IDH mutations promoted glioma cell metastasis and resistance to chemotherapy through up-regulation of the HIF-1α signaling pathway (49). IDH mutations also play an important role in blocking cell differentiation. Mutant IDH blocks hepatocyte differentiation by inhibiting the HNF-4α pathway (50). Other studies have shown that high levels of 2-HG caused by mutations in IDH can inhibit histone and DNA demethylases, resulting in hypermethylation of histones and DNA which eventually blocks cell differentiation (51, 52). Interestingly, there is no significant difference in the median overall survival rate of intrahepatic cholangiocarcinoma patients with mutant or WT IDH (53). In general, mutated IDH catalyzes the production of high levels of 2-HG, which has multiple effects including the inhibition of α-KG-dependent prolyl hydroxylase, which leads to the accumulation of HIF-1α in cells. This results in the induction of HIF-1α target genes that influence growth, migration, differentiation and angiogenesis as well as cell apoptosis (7), ultimately promoting tumor onset and progression (see Figure 1).

## HDACs, HATs, and Their Mutations in Cancer

Histone acetylation is an important epigenetic modification that mainly occurs in the N-terminal region of the histone tail. This modification weakens the binding between histones and DNA, which relaxes the chromatin and enhances gene expression (54). Histone acetyltransferases (HATs) mediate the acetylation in histones, whereas histone deacetylases (HDACs) catalyze the removal of acetyl groups from histones. The HATs are mainly divided into five major families, including GCN5/PCAF, MYST, TAFII250, CBP/p300, and SRC (55). The HDACs are divided into four classes. Class I HDACs include HDAC1, HDAC2, HDAC3, HDAC8; class II HDACs are further divided into two groups, class IIa (HDAC4, HDAC5, HDAC7, HDAC9) and class IIb (HDAC6, HDAC10); class III contains SIRT1-7; and class IV contains one enzyme, HDAC11 (56, 57). Class I, II, and IV HDACs are all Zn^2+^-dependent enzymes, while class III HDACs do not show any sequence similarity to the other three classes and depend on NAD+ as a co-factor (56, 57). By reversing the histone acetylation status, HDACs mostly regulate the expression of tumor suppressor genes (4). The dysregulation of HATs and HDACs is correlated with the occurrence and development of various diseases, including cancer.

Mutations in genes encoding HDACs are associated with the progression of tumors, owing to the abnormal transcription of key genes that regulate important cellular functions such as cell proliferation, cell cycle regulation and apoptosis. Some studies have shown that HDACs are mutated in certain cancers. For example, somatic mutations of HDAC1 were detected in ~8% of dedifferentiated human liposarcomas, and the dysfunction of HDAC2 expression caused by a frame-shift mutation was investigated in human epithelial cancers and in colorectal cancer (58). Table 1 summarizes some of the mutated sites in HDAC2, which may correlate with the development and progression of tumors. However, most of the mutations in HDACs have not been studied and require further investigation. In addition to the discovery that HDACs are mutated in a variety of cancers, there have been many reports in recent years that the HAT CREBBP somatic mutations are more frequent in lymphomas, lung cancer, urothelial carcinoma, and other human tumor types. Jiang et al. reported that somatic mutations in CREBBP occur in 6.4–22.3% of patients with DLBCL and 30.8–68% of follicular lymphoma. Their findings suggest that CREBBP mutation can promote lymphomagenesis *in vivo* (59). Similarly, the results of Jia et al. showed that *CREBBP* acts as a tumor suppressor gene, and its inactivating mutations can promote tumorigenesis of pre-neoplastic neuroendocrine cells and accelerate small cell lung cancer in the autochthonous mouse model (60). The above results suggested that the mutations in HATs and HDACs, although relative low in frequency, might also be involved into the carcinogenesis in various tumors.

## Inhibitors Targeting Mutations of Epigenetic Regulatory Enzymes

### Inhibitors Targeting DNMT Mutants

The DNMT inhibitors 5-azacytidine and decitabine (5-aza-2′-deoxycytidine) have already been approved by the FDA (3). These inhibitors are nucleoside analogs which are incorporated into DNA and then covalently trap DNMTs. The results of research by Xu et al. showed that 5-azacytidine might be a suitable drug for the treatment of AML with DNMT3A mutations (8). In a study comparing small molecule inhibitors of DNMT3A R882H, compound 9 (dichlone) displayed superior efficacy, indicating its potential for targeting mutant DNMT3A (61). Interestingly, a recent study showed that targeting DOT1L, a histone lysine methyltransferase without a SET domain, also has an obvious antitumor effect in DNMT3A mutant leukemia. Rau et al. found that the DOT1L inhibitors SYC-52221 and EPZ004777 decreased tumor cell proliferation and induced cell apoptosis, cycle arrest and terminal differentiation in DNMT3A mutant cell lines in a dose- and time-dependent manner (62). Furthermore, they reported that the DOT1L inhibitor EPZ5676 showed promising efficacy in a nude mouse xenograft model of AML with mutant DNMT3A (62). Since pharmacological inhibitors of DOT1L have been tested in clinical trials, DOT1L may be an indirect therapeutic target for the treatment of AML with DNMT3A mutations. These results suggesting a novel approach for treating patients with DNMT3A mutations.

For the TET2, although inhibitors targeting TET2 mutations have not yet been developed, the results of Bejar et al. indicated that cells in MDS patients with TET2-deficient are more sensitive to azacitidine treatment, and this suggests that patients with MDS carrying the TET2 mutation can improve their response to hypomethylating agents (63). However, the detailed mechanisms mediating this process need further study.

### Inhibitors Targeting EZH2 Mutants

Recently, studies have shown that small molecule inhibitors can effectively target tumors driven by EZH2 mutations. Knutson et al. have reported that the SAM-competitive EZH2 inhibitor EPZ005687, which is highly selective for EZH2 over other methyltransferases, significantly reduced the viability of lymphoma cell lines carrying the EZH2 Y641 and A677 mutations (64). McCabe et al. discovered that GSK126 is a SAM-competitive small molecule inhibitor of EZH2 methyltransferase activity that efficiently and selectively reduced H3K27me3 levels and reactivated the silenced target genes of polycomb repressive complex 2 (PRC2) (41). Their results also revealed that GSK126 effectively inhibited the proliferation of EZH2-mutant diffuse large B-cell lymphoma (DLBCL) cell lines and retarded the growth of EZH2-mutant DLBCL xenografts in mice (41). In addition, EPZ-6438, another selective inhibitor of EZH2, exerted potent antitumor activity against EZH2-mutant non-Hodgkin's lymphoma (65). Also, CPI-1205, an orally available selective inhibitor of EZH2, killed cells in both EZH2-WT and EZH2-mutant B-cell non-Hodgkin's lymphoma by altering PRC2 target gene expression in a dose- and time-dependent manner (5). All of the above inhibitors markedly reduced the high level of H3K27 trimethylation caused by EZH2 mutations, indicating that inhibition of EZH2 methyltransferase activity may be an effective way of treating EZH2 mutant lymphomas. EPZ005687 is currently in preclinical research, whereas GSK126, EPZ-6438 and CPI-1205 are under phase I/II investigation to assess their efficacy in patients with non-Hodgkin's lymphoma and certain solid tumors (5). In view of the high rate of EZH2 mutation in certain cancers, the application of these inhibitors in the clinic is expected to be successful in the future.

However, in addition to focusing on the effects of the EZH2 inhibitor itself on EZH2 mutant enzymes, we also need to consider the use of EZH2 inhibitors in synthetic lethality. Recently, targeting chromatin deficiency in cancer based on synthetic lethality has been used in cancer treatment. Synthetic lethality defines a relationship between two genes, where the loss of either gene is compatible with cell viability, but the loss of both genes causes cell death. Morel *et al*. summarized that the deficiency of SMARCB1, ARID1A, SMARCA4, and PBRM1, which constitute the chromatin remodeling complex SWI/SNF subunit, led to an EZH2 oncogenic dependence in tumor cells, and pharmacological EZH2 inhibitors such as tazemetostat induced dramatic tumor shrinkage in these subunits-deficient tumors (66). Therefore, synthetic lethality strategy may pave the way to potential epigenetic drugs targets.

### Inhibitors Targeting IDH Mutants

Inhibitors targeting mutant IDH enzymes have also been widely investigated. In preclinical studies, it is reported that inhibitors targeting mutated forms of IDH1 and IDH2 can inhibit the growth of glioma cells and induce the differentiation of primary human IDH mutant AML cells *in vitro* (67). Encouragingly, clinical studies of inhibitors targeting mutated IDH have entered the phase I stage, and two inhibitors have been approved by the FDA for clinical use (68). For example, enasidenib (AG-221), a novel-specific small molecule inhibitor targeting mutant IDH2, was approved by the FDA in August 2017 for the treatment of relapsed AML (69). Another novel specific small molecule inhibitor, ivosidenib (AG-120), was approved by the FDA in July 2018 for clinical treatment of relapsed and refractory AML. Ivosidenib targets IDH1 with a mutation at the R132 site (see Figure 1) (70). Three other small molecule inhibitors, AGI-881, IDH305, and FT-2102, are currently in phase I clinical trials. AGI-881 is a non-specific small molecule inhibitor which can target the mutant forms of both IDH1 and IDH2, whereas IDH-305 and FT-2102 target mutant IDH1 (68). These inhibitors prevent the reduction of α-KG to 2-HG by binding to the active site of the mutated IDH enzyme. High levels of 2-HG can inhibit DNA and histone demethylation, leading to hypermethylation. Borodovsky et al. have demonstrated that hypomethylating agents strongly induce differentiation, reduce colony formation ability, and suppress the growth of IDH mutant cells *in vivo* (71). Therefore, inhibitors targeting DNA and histone modifications may have potential therapeutic value. The DNA methyltransferase inhibitors decitabine (DAC) and 5-azacytidine have been approved by the FDA for clinical application and may have a therapeutic effect on tumors caused by IDH mutations (72). These findings also suggest that there is crosstalk among different epigenetic regulatory enzymes. In contrast to IDH mutation inhibitors, studies on inhibitors targeting SDH and FH mutations are currently lagging behind, which may lay the foundation for the development of new anti-tumor drugs.

## Conclusion and Perspectives

DNA methylation and histone modification are common epigenetic changes in eukaryotes, and the dysregulation of epigenetic regulatory enzymes is closely related to the onset and progression of various types of cancer. Mutations, especially gain-of-function mutations, may be responsible for some changes in epigenetic enzyme activity. Mutant epigenetic regulatory enzymes, and mutant forms of IDH that affect epigenetic changes, can enhance the ability of cancer cells to proliferate, migrate and form colonies. Therefore, these mutations are closely related to tumor onset and progression. Some inhibitors that specifically target the mutant forms of epigenetic regulatory enzymes and IDH have now entered clinical trials. The potential therapeutic effects of these inhibitors on tumors caused by mutations are summarized in Figure 1.

Many of the mechanisms by which mutations cause changes in the activity or function of epigenetic regulatory enzymes are not fully understood. Elucidation of these mechanisms may drive our understanding of the characteristics of different tumors. Further research into drugs that target these mutant enzymes will also accelerate the process of individualized treatment of tumors.

## Data Availability

Publicly available datasets were analyzed in this study. This data can be found here: http://www.cbioportal.org.

## Author Contributions

MH contributes to draft manuscript and analysis the data. LJ contributes to draft manuscript. WL contributes to analysis the data. LW and WC contribute to design and draft the manuscript.

### Conflict of Interest Statement

The authors declare that the research was conducted in the absence of any commercial or financial relationships that could be construed as a potential conflict of interest.
